# Distributional reinforcement learning in prefrontal cortex

**DOI:** 10.1038/s41593-023-01535-w

**Published:** 2024-01-10

**Authors:** Timothy H. Muller, James L. Butler, Sebastijan Veselic, Bruno Miranda, Joni D. Wallis, Peter Dayan, Timothy E. J. Behrens, Zeb Kurth-Nelson, Steven W. Kennerley

**Affiliations:** 1 Department of Experimental Psychology, University of Oxford, Oxford, UK; 2 Department of Clinical and Movement Neurosciences, University College London, London, UK; 3 Wellcome Trust Centre for Human Neuroimaging, University College London, London, UK; 4 Institute of Physiology and Institute of Molecular Medicine, Lisbon School of Medicine, University of Lisbon, Lisbon, Portugal; 5 Department of Psychology and Helen Wills Neuroscience Institute, University of California Berkeley, Berkeley, CA USA; 6 Max Planck Institute for Biological Cybernetics, Tübingen, Germany; 7 University of Tübingen, Tübingen, Germany; 8 Wellcome Centre for Integrative Neuroimaging, University of Oxford, John Radcliffe Hospital, Oxford, UK; 9 Sainsbury Wellcome Centre for Neural Circuits and Behaviour, University College London, London, UK; 10 Google DeepMind, London, UK; 11 Max Planck University College London Centre for Computational Psychiatry and Ageing Research, University College London, London, UK

**Keywords:** Reward, Decision

## Abstract

The prefrontal cortex is crucial for learning and decision-making. Classic reinforcement learning (RL) theories center on learning the expectation of potential rewarding outcomes and explain a wealth of neural data in the prefrontal cortex. Distributional RL, on the other hand, learns the full distribution of rewarding outcomes and better explains dopamine responses. In the present study, we show that distributional RL also better explains macaque anterior cingulate cortex neuronal responses, suggesting that it is a common mechanism for reward-guided learning.

## Abstract (web summary)

Muller et al. show that some neurons in the cortex learn faster from better-than-expected outcomes compared to worse-than-expected ones; others do the converse, resulting in simultaneous optimism and pessimism, as predicted by distributional reinforcement learning.

## Main

The prefrontal cortex (PFC) is critical for learning and decision-making^1–6^. RL offers a computational framework for understanding learning and decision-making processes^7^ and explains many neural responses throughout the PFC^8,9^. ‘Classic’ RL models^7,10^ learn to predict the expectation—or mean—of the distribution over possible rewarding outcomes after a stimulus or action. However, by learning only the expected reward, some knowledge of the underlying reward distribution, which may be important for risk-sensitive decision-making, is lost. Furthermore, as all neurons learn to predict the same expected reward, the classic RL framework is unable to account for substantial diversity in reward-related responses across PFC neurons^8,11,12^.

A recent modification to classic RL—distributional RL—learns the full reward distribution and offers a candidate an explanation for neuronal diversity^13–15^. Unlike classic RL models, in distributional RL different neurons learn to predict different parts of the reward distribution. Some neurons encode value predictions above the mean of the reward distribution and others below the mean—referred to as optimistic and pessimistic neurons, respectively. Thus, across the population of neurons the full distribution of possible rewards is encoded and neuronal diversity is predicted. By explaining such diversity, distributional RL better explains responses of midbrain dopaminergic neurons^15^—famously known to encode reward prediction errors (RPEs) that drive learning of reward predictions^16^.

The PFC is engaged during risk-sensitive decisions^17^ and encodes a diversity of learning- and decision-related computations^8,18,19^, including RPEs^8,20^, temporal scales and learning rates^8,21–23^. Given this, and that PFC receives dopaminergic input^24–26^, we examined whether distributional RL explains reward responses in primate PFC in two different decision tasks. In the first dataset, we found key signatures of distributional RL analogous to those shown in mouse dopamine neurons^15^. In the second dataset, we observed a previously untested implication of distributional RL: that there are asymmetries in the rates of learning from better- versus worse-than-expected outcomes.

In the first dataset, we tested three key predictions of distributional RL, replicating the three predictions of Dabney et al.^13^. The first prediction is that different neurons carry different value predictions, varying in their level of optimism. The second prediction is that different neurons have different relative gain factors—or asymmetries—for positive versus negative RPEs. As the different value predictions arise from the different relative gain factors, the third prediction is that these two forms of diversity correlate^15^. Given that RPE-coding neurons are required to test the predictions of distributional RL, we limited our analysis to RPE-coding neurons, that is, those that encode probability at choice and feedback but with opposite signs^8^ (Methods).

**Fig. 1:**
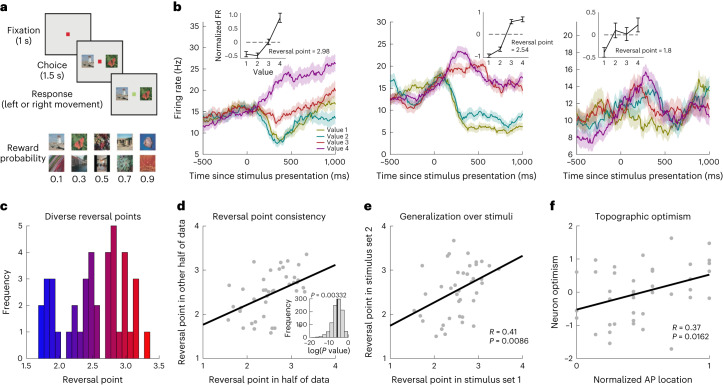
Diverse optimism in value coding across ACC neurons. **a**, Top, on each trial, subjects chose between two cues of neighboring probability value. Bottom, each probability value could be denoted by two stimuli, resulting in two stimulus sets (see ref. ^8^ for task details). **b**, Example responses from three separate neurons demonstrating different levels of optimism. In each plot the mean firing rate is plotted as a function of time and split according to the chosen value (probability) level. There are four chosen values (0.3–0.9 probability) because subjects rarely chose the 0.1 probability level (choice accuracy was at ceiling: 98%). Insets demonstrate that the firing rate is a nonlinear function of value. Mean firing rate (*z*-scored across trials) in a 200- to 600-ms window after cue onset is plotted as a function of the four values. Reversal points are the interpolated values at which there is 0 change from the mean firing rate, an index of nonlinearity. Shaded regions and error bars denote s.e.m. **c**, Histogram showing a diversity of reversal points across ACC RPE-coding neurons. Coloring denotes optimism as defined by reversal point, with red being more optimistic. **d**, Scatter plot showing reversal points estimated in half of the data strongly predicted those in the other half. Each point denotes a neuron. Inset, log(*P* values) of Pearson’s correlation between 1,000 different random splits of the data into independent partitions. Across partitions, the mean *R* = 0.44 and geometric mean of the *P* values was *P* = 0.003 (black line). Bootstrapping to obtain a summary *P* value was also significant (*P* < 0.01). **e**, Scatter plot showing reversal points estimated in stimulus set 1 strongly predicted those in stimulus set 2 (*R* = 0.41, *P* = 0.009). Each point denotes a neuron. **f**, AP topographic location of the neuron predicted its reversal point, with more anterior ACC neurons being more optimistic (*R* = 0.37, *P* = 0.016). As we had two independent noisy measures of each neuron’s optimism (reversal point and asymmetry; Fig. 2), we used the mean of the two measures (after *z*-scoring them), which we call ‘neuron optimism’. Neuron optimism is plotted against the normalized AP locations within ACC. The normalization ensures that, for example, the most anterior portion of the ACC in one animal corresponds to that in the other. Each point denotes a neuron. See Extended Data Fig. 6 for further analyses.

**Extended Data Fig. 1:**
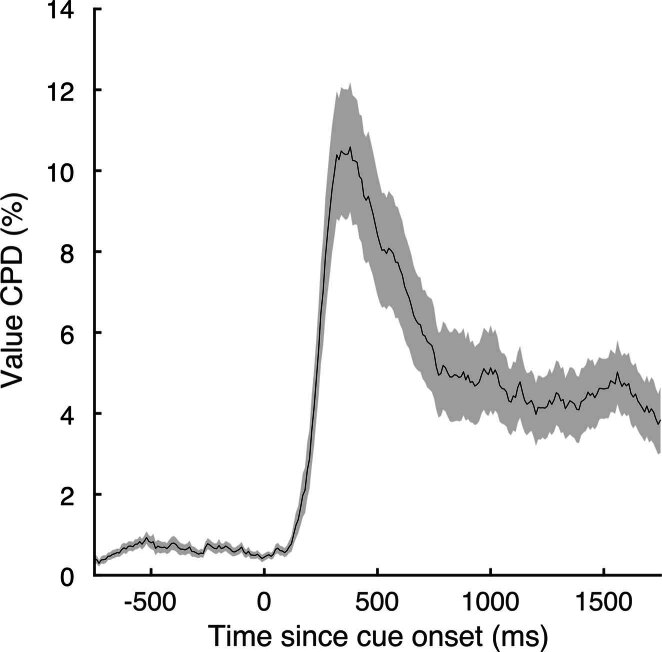
Timeseries demonstrating value-related variance. The mean across neurons of the coefficient of partial determination (CPD) for value (cued probability) over time, following cue onset. The CPD measures how much variance in each neuron’s firing is explained by a given regressor (see below). This timeseries validates the 200–600 ms post-onset window that we used in order to match that used in Dabney, Kurth-Nelson et al.^15^, because that window very closely matches the peak value-related coding in the timeseries (as in Kennerley et al.^8^). We therefore used 200–600 ms and did not try any other time windows in order to avoid any possible p-hacking. Nonetheless we note that in a window that captures this peak, defined as when the CPD is higher than two thirds of the maximum CPD (270–620 ms), the core correlation between reversal point and asymmetric scaling was significant, *R* = 0.38, *P* = 0.019. Shaded region is the SEM across neurons. Note, as in Kennerley et al.^8^, the CPD for regressor *X*_*i*_ is defined by ${{CPD}(X}_{i})=\left[{SSE}\left({X}_{-i}\right)-{SSE}\left({X}_{-i},{X}_{i}\right)\right]/{SSE}\left({X}_{-i}\right)$, where *SSE*(*X*) is the sum of squared errors in a regression model that includes a set of regressors *X*, and ${X}_{-i}$ is a set of all the regressors included in the model except *X*_*i*_. The CPD for *X*_*i*_ is more positive if *X*_*i*_ explains more variance in neuronal firing.

**Extended Data Fig. 2:**
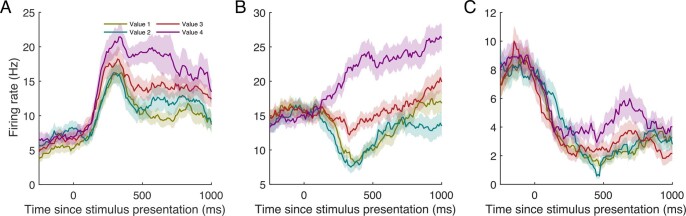
Reason for Z-scoring to fit the reversal points. **a–c**) Three example neurons’ firing rate plotted as a function of time since cue onset, and split according to the four value levels, showing that some neurons increase their firing relative to baseline pre-stimulus firing rate for all reward levels (**a**), others increase or decrease it depending on the reward level (**b**) and others decrease it for all reward levels (**c**). Shaded regions denote SEM. The reason for Z-scoring in our data is as follows. In dopamine neurons, it appears that any firing rate deviation from baseline activity (that is, pre-stimulus onset activity) is signalling a reward prediction error. This is not true for cortical neurons, which may, for example, increase (as in **a**) or decrease (**c**) their firing to all reward levels. If this is the case, then deviation from baseline cannot be assumed to denote an RPE. That some neurons either increase or decrease their firing to all reward levels is indicative of the heterogenous coding schemes evident in PFC neurons. Given this, we can isolate the component of the activity that is associated with RPE by calculating z-scores, and using deviation from mean firing to capture the same effect and compute reversal points. Therefore our reversal point measure captures, for each neuron, the relative differences in responses to different reward levels (that is, the non-linearity) that indicates optimism, rather than being affected by overall shifts in firing. These reversal points, that is, the value at which the neuron firing reverses from below to above the mean firing in the epoch, are an index of neuron optimism; the higher the reversal point, the more optimistic the neuron, and neurons with reversal points above 2.5 are optimistic and below 2.5 are pessimistic (Methods).

To test the first prediction, we examined responses to reward-predicting cues in neurons from three PFC regions implicated in learning and decision-making^8,9,19^: the lateral PFC (LPFC, *n* = 257), the orbitofrontal cortex (OFC, *n* = 140) and the anterior cingulate cortex (ACC, *n* = 213). Two non-human primates (NHPs, *Macaca mulatta*) were presented with a choice of two value-predicting stimuli, which varied in the probability of receiving a fixed magnitude reward^8^ (Fig. 1a). There were four possible choice pairs. Subjects experienced these option pairs thousands of times, virtually always selecting the higher probability stimulus, hence choice value was equivalent to the higher probability option^8^. To test for diversity in value estimates across neurons, we indexed optimism using a measure termed the ‘reversal point’^15^. For each neuron, on each trial, we computed the mean firing rate in a window 200–600 ms after stimulus and subtracted from this the mean firing rate in this window across all trials, to isolate the RPE response (Extended Data Figs. 1 and 2 and Methods). As expected if neurons encode diverse value estimates^15,27^, we observed diverse nonlinearities in individual neurons’ firing rates as a function of reward (Fig. 1b). We indexed this with the reversal point^15^, which is the interpolated cue value at which the mean subtracted firing rate reversed from above to below the mean firing rate (Fig. 1b). In classic RL, the reversal point for all neurons is the mean of the value distribution (that is, 2.5 in this dataset), up to noise. By contrast, in distributional RL, there is genuine diversity between cells, with optimistic versus pessimistic reversal points: optimistic neurons have values above the mean and pessimistic neurons below the mean.

**Extended Data Fig. 3:**
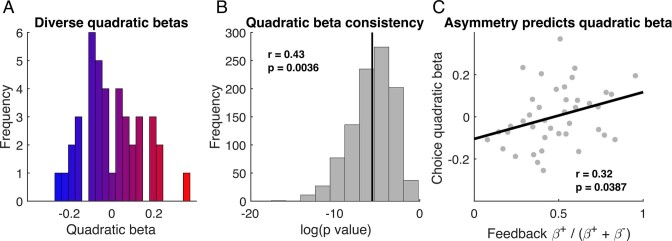
Results hold with a different measure of non-linearity at choice. **a**) Histogram showing diverse quadratic betas. **b**) Histogram showing the log p-values for consistency of these quadratic betas across partitions and the corresponding geometric mean. **c**) Pearson correlation between asymmetric scaling and quadratic betas.

**Extended Data Fig. 4:**
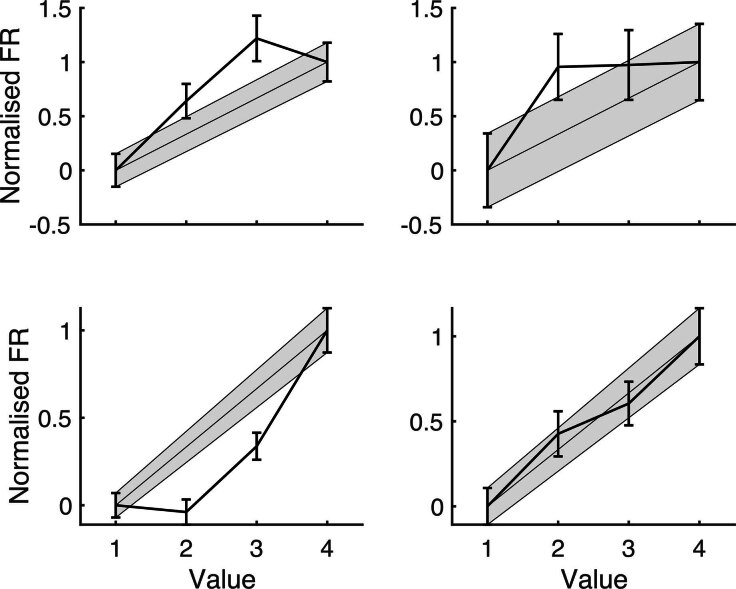
Simultaneous diversity within session. Four simultaneously recorded cells from the session with most reward-sensitive cells (9 in total), demonstrates there is diversity in optimism even within a session. Across cells, responses to middle value levels are both above and below the linear interpolation between lowest and highest values’ responses. Mean normalised firing is plotted for each of the 4 value levels. Error bars denote SEM. Firing rates are normalised such that responses to value 1 and 4 have mean firing rate 0 and 1, respectively. Normalisation allows comparison across cells of responses to middle value levels. Responses to value 2 across the 9 simultaneously recorded cells were significantly diverse; ANOVA rejected the null hypothesis that across cells the value 2 responses were drawn from the same mean (*F*(8,405) = 3.56, *P* = 0.0005). The same was true for responses to value 3 (*F*(8,441) = 2.16, *P* = 0.0291). This diversity was also present when including all cells in the analysis (value 2: *F*(40,1658) = 3.82, *P* = 2.74 × 10^−14^, and value 3: *F*(40,1842) = 4.73, *P* = 4.99 × 10^−20^), and in individual subjects (first animal: value 2; *F*(11,516) = 3.18, *P* = 0.0006, value 3; *F*(10,520) = 3.61, *P* = 0.0001; second animal: value 2; *F*(29,1142) = 3.92, *P* = 2.6 × 10^−11^ value 3; *F*(29,1322) = 5.47, *P* = 1.7 × 10^−18^).

We observed diversity in reversal points across the population of 41 RPE-coding neurons in the ACC (Methods), with both optimistic and pessimistic neurons (Fig. 1b,c). To determine whether this diversity was due to noise, we measured the reversal point independently in the two separate halves of the trials. These two independent measurements were strongly correlated (*R* = 0.44, *P* = 0.003 by Pearson’s correlation; Fig. 1d), suggesting genuine diversity in the reversal point across neurons. Diversity was further evidenced by demonstrating significant diversity across neurons in a different measure of the nonlinearity (Extended Data Fig. 3 and Methods) and in the relative normalized responses to the two middle-value levels (Extended Data Fig. 4).

**Extended Data Fig. 5:**
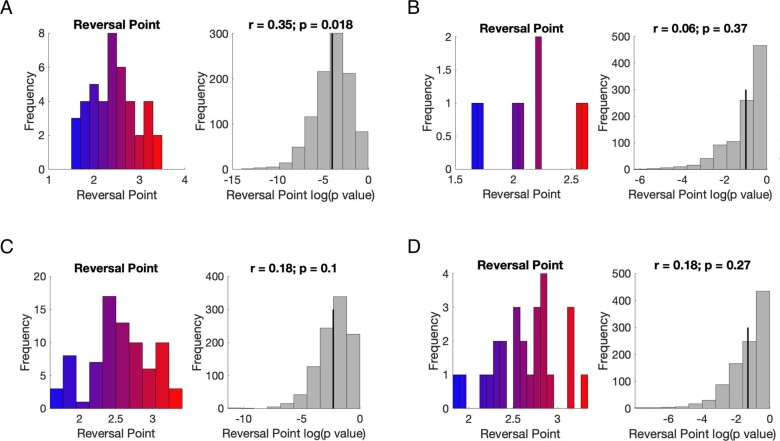
Lack of diversity in OFC and LPFC. We additionally ran analyses in all reward selective neurons (as opposed to only RPE-selective neurons) in OFC and LPFC as an exploratory analysis to assess whether consistent diversity was present when more neurons entered the analysis, since these regions have a smaller proportion of RPE-selective neurons compared to ACC. Same analysis as in Fig. 1, but for OFC and LPFC on all reward-selective neurons or RPE selective neurons. We applied exactly the same criteria and analyses to these brain regions as we did in ACC. As in Fig. 1, we computed the Pearson correlation for each of 1000 independent data partitions, and calculate the mean and geometric mean of the *R* and p-values, respectively. The coloured (left) histograms are the distributions of the reversal points, and the grey (right) histograms are the log(p-values) from the correlations. **a**) OFC reward-selective neurons. **b**) OFC RPE-selective neurons. **c)** LPFC reward-selective neurons. **d**) LPFC RPE-selective neurons. With the exception of the reward-selective neurons in OFC (**a**), none of these analyses were significant. Moreover, when we compared the diversity of these reward-selective neurons in OFC (**a**) across stimulus set (that is, Fig. 1e analysis), the correlation between stimulus set 1 and 2 was not significant (R = 0.15; P = 0.35). This may suggest the diversity in these OFC neurons is due to, for example, stimulus-selectivity, whereby some neurons are selective for stimuli coding the edges of the reward distribution, which could appear as optimism/pessimism in a given stimulus set, but does not generalise across stimulus set as would be expected from diversity related to value. The RPE-selective neurons had no consistent diversity, and as RPE selectivity is a requirement to test further predictions of distributional RL, we did not look for further distributional RL signatures in these brain regions.

Neurons in the OFC and LPFC exhibited lower RPE selectivity (7 of 140 = 6% (OFC) and 26 of 257 = 10% (LFPC), versus 41 of 213 = 19% in ACC), and there was no evidence for consistent diversity in reversal points in RPE-selective neurons in these regions (Extended Data Fig. 5 and Methods). It is possible that the lack of consistent diversity in the OFC and LPFC is the result of these regions having a smaller proportion of RPE-selective neurons, preventing strong claims in favor of or against distributional RL in these regions. Nevertheless, ACC RPE-selective neurons exhibited strong diversity in reversal points, a requirement for testing further predictions of distributional RL. The remainder of our analyses are therefore focused on the ACC.

Distributional RL predicts that reversal diversity is a signature of distributional coding over value, not over cue stimulus features. A neuron tuned to the sensory features of the cue predicting value 4 would appear as an optimistic neuron in our analysis, even though it may not be optimistic in general. Fortunately, the experiment included two different stimuli for each value level. We correlated the reversal point estimated in one stimulus set with that in the other and found optimism in the ACC generalized over stimulus sets (*R* = 0.41, *P* = 0.009 by Pearson’s correlation; Fig. 1e). This confirmed that diversity in ACC reversal points was not explained by tuning to specific stimulus features.

**Extended Data Fig. 6:**
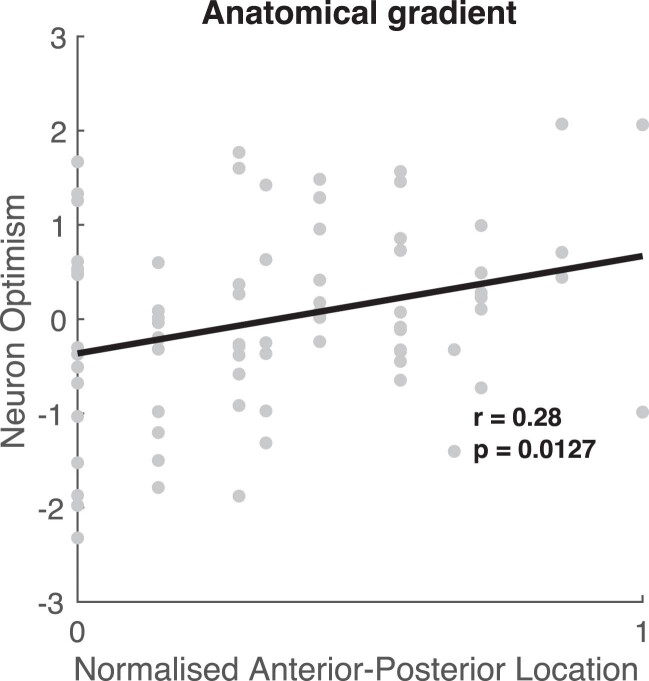
Anatomical gradient in a different subset of probability selective neurons. Here we test for the anatomical gradient across those neurons that were probability selective at choice, but did not meet the criteria for RPEs. This analysis is to test the robustness of the gradient result by assessing whether it replicates in an independent set of neurons. The neuron optimism of these neurons is measured using the reversal point. We present this data here to supplement the gradient analyses in Fig. 1, but note that it is less clear what the predictions of distributional RL are for these non-RPE neurons, and so it is unclear exactly what the reversal point means in these neurons. Nevertheless we present this result to demonstrate the gradient of the reversal point measure replicates in an independent set of neurons (*R* = 0.28, *P* = 0.013, by Pearson correlation).

How distributional computations are supported across brain regions, such that optimistic neurons in one region communicate with those in another, is an intriguing open question^15,28^. Inspired by the topographic organization of learning rates in the ACC^23^, one solution is for topographic organization of degrees of optimism. We tested for such organization and found that the anterior–posterior (AP) location within the ACC predicted optimism, such that more anterior neurons were more optimistic (*R* = 0.37, *P* = 0.016 by Pearson’s correlation; Fig. 1f and Extended Data Fig. 6). Furthermore, as these spatial scales are available to functional magnetic resonance imaging (MRI) and brain stimulation, topography also offers a route to noninvasive measurement and manipulation of distributional representations.

**Fig. 2:**
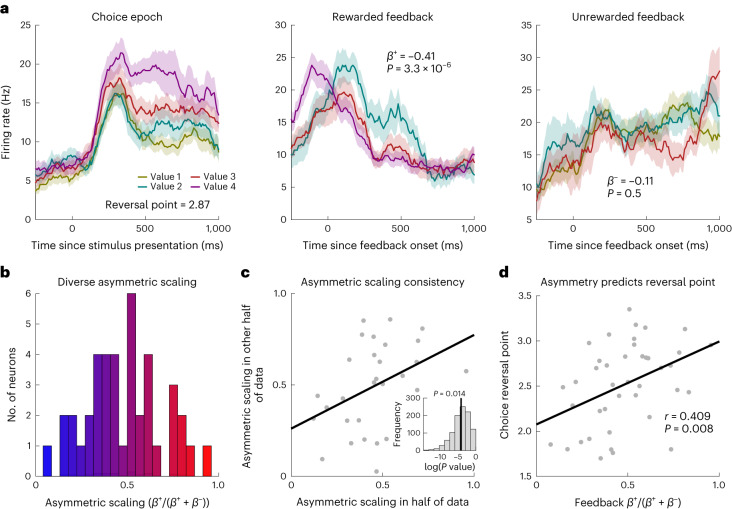
Diverse asymmetric scaling of RPEs predicts optimism. **a**, An example neuron’s responses at each of the task epochs: choice, feedback on rewarded trials and feedback on unrewarded trials. *β*^+^ and *β*^−^ are betas corresponding to the scaling of positive and negative RPEs. Betas are calculated on the mean firing rate in a 200- to 600-ms window after feedback. Error bars denote s.e.m. Note that, for rewarded and unrewarded trials, we do not display the lowest and highest value levels, respectively, owing to a small number of trials giving unreliable traces. **b**, Histogram showing a diversity of asymmetric scaling across ACC RPE neurons. Coloring denotes optimism as defined by asymmetric scaling, with red more optimistic. **c**, Same format as Fig. 1d but for asymmetric scaling consistency: mean *R* = 0.32, *P* = 0.014 across data partitions. Each point denotes a neuron. **d**, Asymmetric scaling estimated at feedback predicted reversal point at choice: *R* = 0.41, *P* = 0.0079. Each point denotes a neuron.

The second prediction of distributional RL is that different cells have different relative gains, or scaling factors, for positive versus negative RPEs^15^. In our task, positive RPEs were (1 − chosen reward probability) on rewarded trials and negative RPEs were (0 − chosen reward probability) on unrewarded trials (Methods). For each neuron, we separately estimated scaling for positive RPEs (*β*^+^), by regressing firing rates against the offer value at feedback on rewarded trials, and likewise scaling for negative RPEs (*β*^−^) on unrewarded trials (Fig. 2a). From these, we computed a single measure (‘asymmetric scaling’) to reflect the asymmetry of positive versus negative RPEs: ${\beta }^{+}/({\beta }^{+}+{\beta }^{-})$. We found diversity in the relative weighting of positive versus negative RPEs across ACC neurons at feedback (Fig. 2b) and this diversity was stable across independent partitions of the data (*R* = 0.32, *P* = 0.014; Fig. 2c).

The third prediction is that optimism (from the first prediction) is correlated with asymmetry in positive versus negative RPEs (from the second prediction); in distributional RL, optimism arises from asymmetric scaling of RPEs. For example, if a neuron upweights—hence learns more—from positive than from negative RPEs, it will learn an optimistic value prediction. We confirmed this prediction in ACC neurons: asymmetry in RPEs at feedback predicted the reversal points at choice (*R* = 0.41, *P* = 0.0079 by Pearson’s correlation; Fig. 2d). This is a specific prediction of distributional RL^15^. Thus primate ACC contains analogs of distributional RL found in rodent dopamine neurons^15^.

**Fig. 3:**
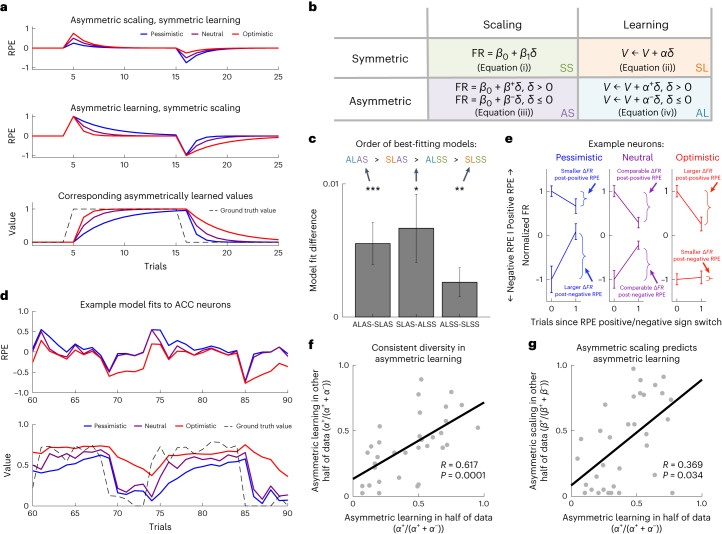
Diverse asymmetric learning. **a**,**b**, Asymmetric scaling and asymmetric learning are both predictions of distributional RL, but are dissociable. Asymmetric scaling reflects differences in the degree to which positive and negative RPEs are scaled to predict firing rate. Asymmetric learning reflects differences in the rate of state value update after positive and negative RPEs (which may or may not be affected by asymmetric scaling). These different learning rates are denoted by *α*^+^ and *α*^−^, respectively. $\delta =r-V$ is the RPE, where *r* is the reward on the current trial and *V* the value. **a**, Simulated examples demonstrating the difference between asymmetric scaling and learning, as governed by the equations in **b**. The top shows predicted RPEs generated by asymmetric scaling with symmetric learning (equations (iii) and (ii)). In this extreme case, the scaling does not impact learning and the learned value would converge on the expectation. The middle and bottom show the converse: RPEs and corresponding values generated by symmetric scaling with asymmetric learning (equations (i) and (iv)). We have presented them in this way to highlight how asymmetric scaling and asymmetric learning can be dissociable phenomena that we can measure separately, not because we do not predict that they are related. On the contrary, we show that they are related in **g**. **c**, Comparing the crossvalidated model fits revealed that a model with both asymmetric learning and asymmetric scaling (ALAS) is the best explanation of the ACC data, and the fully classic (symmetric) model (SLSS) is the worst model of the data. Each bar in the bar graph shows the comparison between a pair of models and is the difference in the *R*^2^ value of the two models being compared. Error bars denote s.e.m. The significance of the differences is determined by paired, two-sided Student’s *t*-tests over neurons: ^*^P ≤ 0.05, ^**^P ≤ 0.01, ^***^P ≤ 0.001. **d**, Example model fits. Top, RPE regressors generated using learning rate parameters fitted to individual neuron data, for three different neurons from the same session. Different levels of optimism can be seen via the different rates at which RPEs tend back toward zero after changes in state value (denoted by the dashed black line in the bottom plot). Bottom, this is reflected in the corresponding values. The pessimistic neuron (shown in blue), for example, is quick to devalue but slow to value. **e**, Example real neuron responses around transitions in the sign of the RPE, from three separate neurons. We used the best-fitting model to define trials when the RPE switched from negative to positive, or vice versa. We then plotted the mean firing rate on that first trial of the switch and the subsequent trial, and observed asymmetries in the rate of change in the firing rate after the first positive versus negative RPE, as predicted by distributional RL in **a**. For example, the (pessimistic) neuron on the left changes its firing rate more following negative than positive RPEs (the slope for negative RPEs is more positive than the slope for positive RPEs is negative), indicating that it has learnt more from negative than from positive RPEs. The converse pattern is true for the (optimistic) neuron on the right. Error bars denote s.e.m. **f**, The per-neuron asymmetry in learning derived from the model, defined as ${\alpha }^{+}/({\alpha }^{+}+{\alpha }^{-})$, estimated in one half of the data predicted that in the other half of the data (*R* = 0.62, *P* = 0.0001), demonstrating that there is consistent diversity in asymmetric learning across the population of neurons, as predicted by distributional RL. **g**, Asymmetric learning and asymmetric scaling positively correlated, consistent with the theoretical proposal that asymmetric scaling drives asymmetric learning (*R* = 0.35, *P* = 0.04 for a correlation between asymmetric learning estimated in the first data partition and asymmetric scaling estimated in the second, and *R* = 0.38, *P* = 0.03 for the converse correlation; average across partitions: mean *R* = 0.37, geometric mean *P* = 0.03).

So far, we had identified neural signatures of distributional RL in a static task where values did not need to be updated. However, many real-world contexts require continuous learning as decision values change. We then turned to a previously untested, strong prediction of distributional RL; in addition to diverse asymmetries in the scaling of positive versus negative RPEs, we expected diverse asymmetries in the rates of learning from positive versus negative RPEs (Fig. 3a,b). Optimistic cells should learn rapidly from positive RPEs and slowly from negative RPEs, and pessimistic cells the opposite. This should be detectable in subsequent RPE responses, because the RPEs are computed using the learned value. After a positively surprising event, the size of positive RPEs in an optimistic cell should decrease sharply because the value prediction is sharply increased, with the converse pattern in pessimistic neurons (Fig. 3a).

**Extended Data Fig. 7:**
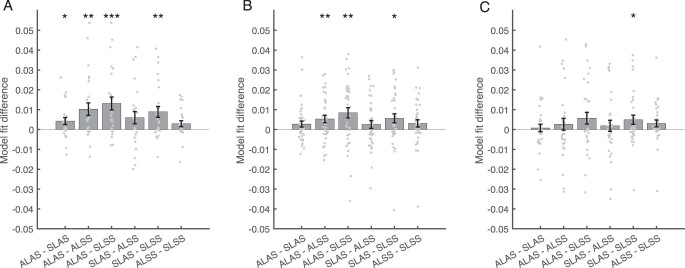
Other brain regions demonstrate some or no evidence for distributional RL. Same format and analyses as Fig. 3c in the main text, and Extended Data Fig. 8. We repeated our model comparison analyses in the other brain regions recorded in this task. These regions are also known to contain reward and prediction error signals, and so we may expect them to carry signatures of distributional RL. We found evidence for distributional RL in caudate (**a**; n = 26 neurons), weak evidence for it in dorsolateral prefrontal cortex (**b**; n = 39), and no evidence for it in putamen (**c**; n = 34). Error bars denote SEM. *P ≤ 0.05, **P ≤ 0.01, ***P ≤ 0.001. However we note that, similar to the first dataset presented in this manuscript, the number of selective neurons in these other regions is smaller than in ACC (which had 94 out of 240 neurons selective; 39%); caudate had 26 out of 115 neurons (23%), dorsolateral prefrontal cortex had 39 out of 187 neurons (21%), and putamen had 34 out of 119 neurons (29%). Furthermore, there were too few neurons selective under the stricter criteria for defining RPE-selective neurons (from Bayer & Glimcher 2005 and used in other parts of this manuscript; Methods), and so we do not analyse the model comparisons in these regions further; caudate (9 out of 115 neurons; 8%), dorsolateral prefrontal cortex (7 out of 187 neurons; 4%), and putamen (11 out of 119 neurons; 9%). We therefore do not wish to make claims about the presence or absence of distributional RL in these regions; rather it is possible the lack of strong evidence for distributional RL in these regions arises from a smaller proportion of neurons that are encoding RPEs.

Exploring asymmetric learning requires a learning task in which the reward structure is dynamic, so that subjects must update their value expectations. In a second dataset, we analyzed single-neuron data in the PFC and striatum from two NHPs (*M. mulatta*) during performance of a well-studied learning task^29,30^. In this task, there were four cues that independently changed in value every five to nine trials (Methods). To maximize reward, subjects needed to update their value estimates of these cues across trials. We identified a significant population of RPE-selective neurons in the ACC (*n* = 94 of 240, 39%), which we used for our analysis (RPE selectivity was defined from a Rescorla–Wagner-based learning model from Miranda et al.^30^; Methods). As in the nonlearning task presented earlier, we found little evidence for distributional RL in other brain regions, again possibly owing to a smaller proportion of RPE-sensitive neurons (Extended Data Fig. 7). Therefore, combined with the fact that we have a hypothesis for distributional RL in the ACC from the first dataset, we focused on the ACC.

We fitted four models to the neuronal responses, specifically to firing rate at outcome. All models were adaptations of the Rescorla–Wagner model, wherein learning of values is driven by RPEs scaled by a learning rate (see Fig. 3b for equations and Methods for details). One model allowed each neuron to have a different, therefore asymmetric, scaling of RPEs, as described in the analysis of the nonlearning task, and symmetric learning from RPEs (that is, governed by equations (iii) and (ii) from Fig. 3b, respectively). One model allowed each neuron to have different learning rates for positive versus negative RPEs, with symmetric scaling (that is, equations (iv) and (i), respectively). One model, classic RL, allowed neither a degree of flexibility (that is, equations (i) and (ii)); one model, fully distributional RL, allowed both (that is, equations (iii) and (iv)). For each neuron, we fitted the learning rate and scaling parameters in a subset of the data (Methods). We then used these parameters to generate RPE regressors for the held-out data in which we assessed the model’s fit to the data (*R*^2^) using tenfold crossvalidation. We compared the different models’ fits to the data using the mean *R*^2^ (across partitions) for each neuron.

**Extended Data Fig. 8:**
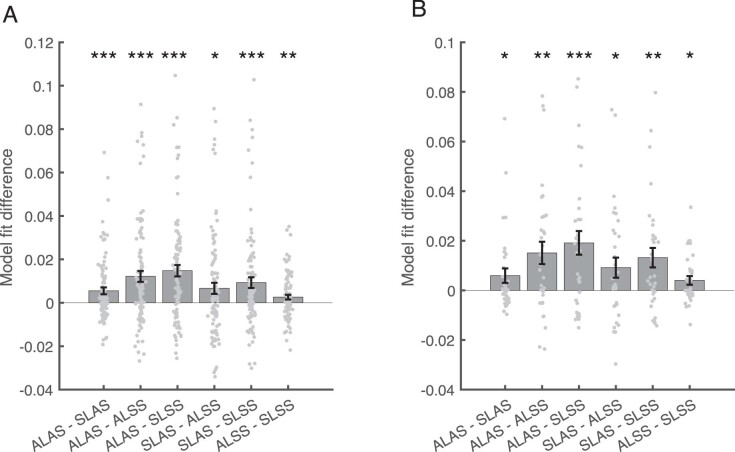
All pairwise model comparisons for asymmetric learning and scaling analyses. Same format as Fig. 3 in the main text. **a**) Bar graphs with all 6 pairwise model comparisons for the 94 neurons defined as selective using the RPE regressors from Miranda et al.^30^ (Methods). ALAS – SLAS, SLAS – ALSS, and ALSS – SLSS are the same as in the main text. *P ≤ 0.05, **P ≤ 0.01, ***P ≤ 0.001. **b**) Same as A but for only those neurons (n = 33) that meet a strict definition for being RPE selective. That is, as defined in Bayer & Glimcher 2005, those neurons that encode reward on the current trial and previous trial but with opposite signs (see Methods for further details).

**Extended Data Fig. 9:**
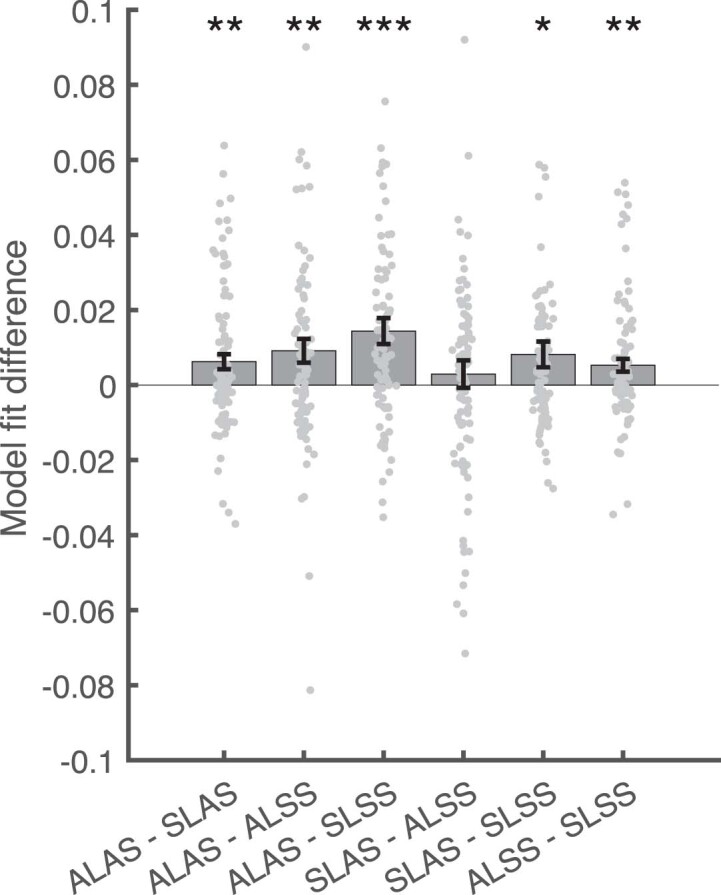
Results are very similar when taking the linear parameters *β*_0_ and *β*_1_ across cross-validated partitions. Same format as Fig. 3 in the main text. In the main text analyses, we re-fit the linear parameters *β*_0_ and *β*_1_ in the test data during cross-validation. As explained in the Methods, this is to isolate our analysis to the asymmetries in scaling, rather than the analysis being impacted by, for example, overall (non-asymmetric) gain. Here we show the same result as in Fig. 3c in the same 94 neurons, but when carrying over the linear parameters (*β*_0_ and *β*_1_) as well as the asymmetric parameters (*S*, *α*^+^ and *α*−) to predict firing rate, and therefore do not re-estimate the linear parameters in the test data. We find that the pattern of results remains the same. Error bars denote SEM. *P ≤ 0.05, **P ≤ 0.01, ***P ≤ 0.001.

We found that the model incorporating both asymmetric learning and asymmetric scaling (fully distributional RL) was the best explanation of the ACC data (Fig. 3c and Extended Data Figs. 8 and 9). This suggests that two learning rates—one for positive RPEs and another for negative RPEs—are better than one in explaining the learning dynamics of ACC neurons.

**Extended Data Fig. 10:**
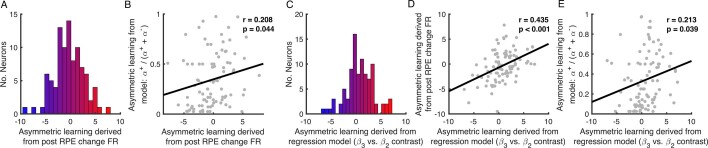
Alternative model agnostic, directly data-derived, measures of asymmetric learning correlate with model fit parameter-derived asymmetric learning. **a**) Similar to the analysis in Fig. 3e, wherein we analysed the neural firing rates around transitions in the sign of the RPE (as defined from the best-fitting model), we also analysed firing rates on the first and second trials following the highest reward option and the lowest reward option (that is, analogous to Fig. 3e but where the x-axis is trial number following the onset of consecutive trials of highest – or lowest – reward level delivered). On these trial types we can be confident that all neurons (regardless of optimism) will have positive and negative RPEs, respectively (because these reward levels are at the extremes of the reward distribution). We observed diversity across the population of neurons in a per-neuron t-score measure, obtained from an unpaired *t-*test testing for differences in firing rate change between the first and second trial following a high reward vs. the same following a low reward. (Note this is the same measure that we used in the main text to provide a per-neuron measure capturing the asymmetries plotted in Fig. 3e, which we correlated with model-derived asymmetric learning.) These t-scores reflect the per-cell significance in rejecting the null hypothesis that there is no difference in firing rate change from the first to second trial receiving highest reward, vs. that on lowest reward. **a** is a histogram of these t-scores, and demonstrates there is significant diversity across the population. **b**) This t-score measure in **a** correlated across neurons with asymmetric learning derived from the best-fitting model (*R* = 0.21, *P* = 0.044). **c**) Additionally, we constructed a regression model to capture asymmetries in the effect of a highest vs. lowest reward level delivered on the previous trial on the current trial’s firing rate. This also captures asymmetries in learning. The regression model was the following: $FR=\,{\beta }_{0}+{\beta }_{1}Rew(t)+{\beta }_{2}HighestRew(t-1)+{\beta }_{3}LowestRew(t-1)$, where *Rew*(*t*) is the reward on the current trial, *HighestRew*(*t*−1) is a binary regressor with value 1 if the previous trial delivered the highest reward level and 0 otherwise, and *LowestRew*(*t*−1) is similarly a binary regressor with value 1 if the previous trial delivered a lowest reward level and 0 otherwise. We then do a [1 -1] contrast for *β*_3_ vs. *β*_2_ to capture differences in the effect of a highest vs. lowest reward delivered on the previous trial on the current trial’s firing rate. This value will be more positive if delivery of the highest reward level on the previous trial decreases the firing on the current trial more than delivery of the lowest reward level increases it (this pattern would be expected from an optimistic neuron). Delivery of the highest reward level is expected to decrease firing on the subsequent trial (captured by *β*_2_) due to the learning induced from positive outcomes: it should suppress subsequent RPEs as the value expectation is now higher (same logic as in Bayer & Glimcher^31^). Similarly, delivery of the lowest reward level is expected to increase firing on the subsequent trial (captured by *β*_3_) due to the learning induced from negative outcomes: it should increase subsequent RPEs as the value expectation is now lower. The [1 -1] contrast testing *β*_3_ vs. *β*_2_ captures the relative differences in these effects and is therefore another index of asymmetric learning: optimistic neurons should be more impacted by the highest reward level compared to the lowest. We found the t-scores of this contrast were also diverse across the population (**c**), correlated with the other data-driven measure described above in **a** and **b** (**d**; *R* = 0.44, *P* < 0.001), and also correlated with asymmetric learning derived from the best-fitting model (**e**; *R* = 0.21, *P* = 0.039). Combining both of these noisy measures from **a** and **c** into a hybrid measure (by averaging the t-scores) gives a summary model-agnostic measure that is also correlated with model-derived asymmetric learning: *R* = 0.25, *P* = 0.016.

To offer intuition into what is being fit, we analyzed neuronal firing around transitions in the sign of the RPE. Some (optimistic) neurons exhibited sharper decreases in firing after positive RPEs than increases in firing after negative RPEs (Fig. 3d,e), as predicted by distributional RL (Fig. 3a). Some other (pessimistic) neurons showed the converse pattern. A per-neuron measure capturing this asymmetry correlated with the asymmetric learning derived from the best-fitting model (*R* = 0.35, *P* = 0.0005; see Extended Data Fig. 10 for further analysis details). Further measures that capture asymmetries in learning—derived directly from the data and therefore not dependent on the modeling—also correlated with model-derived asymmetric learning and showed significant diversity across the population, thereby providing additional evidence for asymmetric learning (Extended Data Fig. 10).

We next analyzed the fitted parameters in a subset of neurons that met a more stringent definition of RPE, that is, those neurons that encode reward on the current and previous trial, but with opposite signs^31^ (Methods). In these 33 neurons, the model fit results all held (Extended Data Fig. 8). To corroborate the model comparisons and to demonstrate that asymmetries in learning in this model were consistent and diverse across neurons, we showed that they were stable across independent partitions of the data (Fig. 3f; *R* = 0.62, *P* = 0.0001, from an across-neuron correlation over data partitions). This was also true for asymmetries in scaling (*R* = 0.58, *P* = 0.0004). These results would be expected only if the asymmetric learning and scaling effects were real. Therefore, different neurons have different relative rates of learning from positive versus negative RPEs: one neuron may rapidly increase its value following positive RPEs but slowly decrease it following negative RPEs, and vice versa for another neuron (Fig. 3d–f). Together, these results provide evidence for a previously untested key prediction of the distributional RL theory.

We next tested for and found a positive relationship between asymmetric scaling and asymmetric learning (Fig. 3g; *R* = 0.37, *P* = 0.03). This result does not preclude additional alternative possible mechanisms for how asymmetries in learning may arise, such as asymmetric synaptic gain^15^. It is, however, consistent with the most straightforward neural implementation of distributional RL: that diversity in scaling causes diversity in learning, because larger RPEs drive larger learning updates.

Distributional RL provides a powerful computational framework that learns the full reward distribution rather than only the expectation, improves performance of artificial agents and explains rodent dopaminergic responses better than classic RL^13–15^. In the present study, we demonstrate that distributional RL also better explains single-neuron responses in the cortex, specifically in primate ACC. We found diverse value predictions that were correlated with diverse asymmetries in RPE scaling—analogs of the results in dopamine. Diversity generalizes over stimulus features, hence it is not an artifact of stimulus feature coding, and lies on an anatomical gradient, which may organize computations across brain regions^15,28^. Finally, we observed consistent diversity across neurons in the asymmetries in their rates of learning from positive versus negative RPEs, marking, to our knowledge, the first test of the dynamic predictions of distributional RL.

The presence of distributional coding in the PFC has several implications. First, it provides a candidate mechanism for how cortical representations of probability distributions over value arise, important for value-based, risk-sensitive decision-making^17,32^. Second, PFC responses are diverse, with different neurons showing different selectivity profiles^11,19,33^. Distributional RL does not explain all of this diversity, although it raises the question of whether similar mechanisms could drive diversity beyond reward prediction. Third, it raises intriguing questions about the relationship between dopaminergic and cortical distributional RL. One possibility is that cortical diversity is simply inherited from topographically organized dopaminergic circuits. Another possibility is that independent distribution-learning mechanisms arise within the PFC as a byproduct of meta-learning^15,34^. Finally, the presence of distributional RL in primate PFC across two different studies suggests that distributional RL may be a ubiquitous mechanism for reward-guided learning.

## Methods

### Dataset 1

#### Task and neural recordings

Results in Figs. 1 and 2 are a re-analysis of the data presented in Kennerley et al.^8^. Full task and recording details can be found there, but we outline the key points relevant to the present study here. All procedures complied with guidelines from the US National Institutes of Health and the University of California Berkeley Animal Care and Use Committee^8^.

Two different sets of two NHPs (rhesus macaques) were used in both dataset 1 and dataset 2. Two is the commonly used number for macaque studies and is standard across virtually all macaque electrophysiology studies. Please note that the data analyzed in the present paper are from two previously collected datasets (datasets 1 and 2). Therefore, no new animals were used in the present study. Subjects in dataset 1 were two males that were aged 5–6 years and weighed 8–11 kg at the time of recording. Although the tasks in these datasets were suited to testing for distributional RL, we also discussed a possible experiment to further test for distributional RL in the Supplementary Information.

The task in Dataset 1 was a two-alternative, forced choice task, in which two rhesus macaques were presented, on each trial, with two stimuli, which they chose between with the use of a joystick movement. After a delay, feedback was delivered. Trials differed in the pair of stimuli presented at the choice phase. Stimuli were drawn from a set of possible stimuli, which denoted different values varying along one of three attributes: probability of reward, magnitude of reward or the amount of effort (lever pulls) required to obtain the reward.

On any given trial, subjects were presented with two stimuli from the same attribute (for example, both probability cues), from the same stimulus set and of neighboring values (for example, subjects chose between 0.9 and 0.7 probability cues, never 0.9 and 0.5), hence the chosen value difference was the same on all trials. For the purpose of the present study, our analyses focused only on probability (not magnitude or effort) trials.

Recordings were made in the ACC (*n* = 213 neurons), OFC (*n* = 140) and LPFC (*n* = 257) (see Figure 6 of Kennerley et al.^35^ for precise locations of recorded neurons).

This dataset is well suited to testing for distributional RL given that recordings were in the ACC, a region known to contain value-related learning signals^8^ and to be important for risk-sensitive decision-making^17^. Furthermore, this dataset is well suited because we can index neural responses to positive and negative RPEs separately^8^ (see below). Indeed, we previously reported^8^ that some neurons in the ACC encode, for example, positive RPEs more strongly than negative RPEs, which is suggestive, broadly speaking, of diversity in RPE coding. In addition to being the most appropriate brain region to test for distributional RL in the cortex, the ACC is also recorded in both this dataset and the other dataset analyzed in this paper (see below).

Note that we present results only from the analyses of probability trials, because these are the only trials in which we can measure asymmetric scaling of RPEs—the probabilistic feedback causes positive and negative RPEs on rewarded and unrewarded trials, respectively (see below). Also note that distributional RL makes predictions at the neural level and so our analyses focused on the neural data.

#### Neuron inclusion criteria and analysis assumptions

Only neurons that encoded RPE were entered into subsequent distributional RL analyses. To meet this criterion, neurons must be probability selective at choice, defined as *P* < 0.05 in linear regression between probability level and mean firing rate on each trial in a 200- to 600-ms window after cue onset. This is the analysis window used throughout the study and was chosen because it matches that used in Dabney et al.^15^ and because there is strong reward-related activity in this time period^8^ (Extended Data Fig. 1). Neurons must in addition encode reward probability at feedback with an opposite sign to that at choice, that is, RPE-selective neurons (see below), as we defined previously^8^. Forty-one ACC neurons (19%) met this criterion; in contrast, only 6% of OFC and 10% of LPFC neurons met this criterion. Furthermore, we did not find significant diversity in the reversal point in OFC and LPFC RPE neurons (Extended Data Fig. 5), which may be the result of a smaller proportion of neurons encoding RPE. Hence, we focused on the ACC for the remainder of the analyses. Although we restricted our analysis to RPE-coding neurons as defined above, we note that neural responses to the onset of the stimuli can be thought of as a prediction error to the cue probability, because it signals whether the current trial offer was better or worse than expected^8^.

We briefly note here that, unlike dopaminergic neurons, in the ACC some neurons’ firing rates have a positive relationship with reward (that is, the firing rate increases as the reward increases) and others negative (that is, the firing rate increases as the reward decreases)^8^. We therefore flipped the firing rates (multiply by −1) of those neurons that are negative, but note that this in fact does not make any difference to the estimation of the distributional RL measures.

Furthermore, as the value differences of the choices were constant (as they are only ever show pairs of stimuli neighboring in value) and their performance was at ceiling^8^ (choosing the higher option on 98% of trials), we had four possible pairs of stimuli (and chosen values) that could be experienced on each trial.

#### Measuring optimism at choice

We indexed the nonlinearity in the firing rate as a function of reward using a measure analogous to that used in Dabney et al.^15^. We measured the ‘reversal point’ of a neuron by estimating the value at which that neuron’s response is the same as (or reverses from positive to negative deviation from) the mean firing rate across trials after the presentation of the value-predicting cue (in the analysis window).

Unlike in dopaminergic neurons, the reversal point here is induced by *z*-scoring the data (mean firing rate in the analysis window after stimulus onset) within a neuron and across trials, and is therefore not exactly the same as the reversal point from baseline (pre-stimulus onset) firing, as used in Dabney et al.^15^. This is necessary because deviation in the firing rate from baseline in cortical neurons does not have the same assumed meaning as it does in dopaminergic neurons. In dopaminergic neurons, it is assumed that positive and negative deviations from baseline firing rate equate to positive and negative RPEs being signaled by that neuron^15,16^. However, in the cortex, many probability selective neurons will, for example, increase their firing rate (relative to the pre-cue baseline) in response to all values (that is, even those at the lowest part of the reward distribution, which ought to elicit negative RPEs even in the most pessimistic neurons). Hence, unlike in dopaminergic neurons, in the cortex an increase in the firing rate relative to the baseline does not necessarily mean a positive RPE (Extended Data Fig. 2). We therefore measured the reversal point for each neuron by *z*-scoring the data in a window after feedback, so that we could compare the measures of optimism across neurons (this *z*-scoring results in neutral neurons having a reversal point of 2.5 and deviations >2.5 and <2.5 indicating optimism and pessimism, respectively). The reversal point is estimated by linearly interpolating between the neighboring negative and positive state values and is defined as the value at which that interpolation crosses no change from the mean firing rate (Fig. 1b). If a neuron is optimistic and thus predicts the highest values in the range of the task, the firing rate to all values but the highest value will be low relative to that of the highest, hence the reversal point will be high (Fig. 1b, left). We used this reversal point measure for consistency with Dabney et al.^15^.

However, we noted that an alternative measure of optimism capturing the nonlinear shape of the neuronal response as a function of reward yielded qualitatively the same results (Extended Data Fig. 3) and was highly correlated with the reversal point. This measure is obtained by fitting the nonlinearity in the firing rate as a function of reward using a quadratic term in linear regression:

1 $${{\mathrm{FR}}}={\beta }_{0}+{\beta }_{1}R+{{\beta }_{2}R}^{2}$$

where FR is the firing rate on each trial and *R* the reward level. *β*_2_ is a regression weight that indexes optimism via the concavity (or convexity) of the function. As expected, this measure of optimism is highly correlated with the reversal point described above (*R* = 0.87, *P* = 4.0 × 10^−37^ by Pearson’s correlation), corroborating that both measures index the nonlinearity in the firing rate as a function of reward.

Such nonlinear responses have recently been shown to arise from normalized RL, wherein rewards are represented by a normalized objective function inspired by a canonical divisive normalization computation^27^. Such normalization may be particularly relevant to cortical neurons. Importantly, it also offers a mechanism for how nonlinear reward coding compatible with distributional RL may arise in a biologically plausible manner and, furthermore, how this may naturally give rise to distributional RL^27^. This work therefore provides a deeper possible explanation and mechanism for how the effect captured by our reversal point and quadratic *β* measures may arise and result in distributional coding.

In terms of what reversal points we expect to see in our data, we noted that, although the probability distribution over value was uniform (each of the four value levels was equally likely to be presented at choice on a given trial), this did not necessarily mean that we expected the measured reversal points to be a uniform distribution. This is because the learned reversal points arising from distributional RL are predicted to correspond to expectiles of the reward distribution (Dabney et al.^15^). Therefore, we did not expect the measured reversal points (in Fig. 1c) to be uniform; we did, however, expect them to exhibit consistent diversity (as shown in Fig. 1d,e).

#### Consistent diversity in optimism at choice

Observing diversity in optimism/reversal point alone is not sufficient, because this would be expected simply by noise. We therefore confirmed that diversity in reversal points was consistent by partitioning the data into independent partitions and testing whether the diversity was consistent across the partitions. We followed the same methodology as Dabney et al.^15^. We estimated the reversal point in a random half of the trials and repeated the estimate in the other half. We did this for each neuron and then correlated the reversal points estimated in one half with those in the other half, obtaining *R* and *P* values for the correlation. If diversity were not the result of random noise, we would expect these independently estimated reversal points to significantly correlate across neurons. To ensure that this correlation is robust across partitions of the data, we repeated this partitioning process 1,000× and took the geometric mean of the *P* values across partitions to obtain a summary *P* value for the analysis. We also obtained a summary *P* value by bootstrapping, wherein we used the random partitions to obtain a *P* value of the correlation coefficient.

#### Asymmetric scaling (RPEs at feedback) analysis

To estimate asymmetry in the scaling of the firing rate as a function of positive versus negative RPEs, we estimated the scaling of positive and negative RPEs on rewarded and unrewarded trials, respectively. This was possible because rewarded trials would always elicit positive RPEs, and vice versa for unrewarded trials. The scaling of the firing rate as a function of, for example, positive prediction error was the regression weight used to scale the positive RPE to predict the firing rate, as in Dabney et al.^15^. The size of the RPE was dependent on the cued probability at choice^8^. The RPE was defined as *r* − *V*, where *r* is the delivered reward and *V* the cued probability value that denotes the expected value of the upcoming outcome. Rewarded and unrewarded trials yielded a reward of 1 and 0, respectively. If, for example, the cued probability were high (0.9), this would elicit a smaller positive RPE on rewarded trials than a low cued probability (0.3), because reward would be more expected (the RPEs in these cases would be: 1 – 0.9 = 0.1 and 1 – 0.3 = 0.7, respectively). In contrast, high cued probabilities would elicit larger negative RPEs on unrewarded trials, because a reward was expected. We therefore estimated the scaling of positive and negative RPEs by regressing the chosen cue probability against the firing rate at feedback, separately for rewarded and unrewarded trials, resulting in the regression coefficients *β*^+^ and *β*^−^ for scaling of positive and negative RPEs, respectively. We used this scaling to compute the optimism of the scaling asymmetry as ${\beta }^{+}/({\beta }^{+}+{\beta }^{-})$. To confirm that the revealed diversity was not simply a result of noise, we performed the same partition-based consistency analysis as we did for optimism at choice.

This measure is analogous to the asymmetric scaling measure used in Dabney et al.^15^, the difference being that, whereas Dabney et al.^15^ measured asymmetric scaling from the cue presentation epoch, we estimated the scaling at a separate task epoch to cue presentation/choice; that is, at feedback time, when RPEs will be elicited after a cued probabilistic reward delivery. Furthermore, we estimated positive and negative RPEs on rewarded and unrewarded trials, respectively.

To test for the relationship between optimism at choice and asymmetric scaling, we regressed choice optimism against asymmetric scaling across neurons. We performed this analysis for those neurons that encode RPEs—in other words, following Kennerley et al.^8^, those neurons that code the cued probability in choice and feedback epochs with opposite signed relationships, and where both feedback epochs (rewarded and unrewarded) had the same sign. In brief, the logic is as follows: an RPE-selective neuron that, for example, increases its firing rate as a function of chosen probability at choice, and therefore has a positive relationship between firing rate and RPE (elicited by the probability cue), should fire less strongly after reward following a high probability cue (because the RPE is smaller), and therefore has a negative relationship between firing rate and probability at feedback. This same negative relationship applies on unrewarded trials, when a larger decrease in firing is elicited on high probability trials because a larger negative prediction error is elicited by lack of reward on high probability trials. Hence, the sign of the relationship between firing rate and probability cue is opposite at choice and feedback for RPE-selective neurons as previously explained in detail^8^. Choice optimism and feedback asymmetric scaling are measured in different trial epochs (that is, choice and outcome), minimizing the likelihood of artefactual correlation.

Note that we did not look for asymmetric learning (see below and main text) in dataset 1. This was because the animals had been overtrained on this task and little to no learning remained at the time of recording. The animals’ behavior was at ceiling (accuracy, defined as selecting the higher value option, was mean 98%, s.e.m. 0.2%). Nevertheless, the brain still computed prediction errors, which we (and Dabney et al.^15^) used to measure distributional RL. It is not fully understood why the prediction errors in static tasks do not drive the same kind of learning as they do in dynamic tasks. Evidently, there is a downstream mechanism that regulates the degree of learning from these signals. Mechanisms such as Bayesian RL (Behrens et al.^36^) or meta-RL (Wang et al.^34^) may be at play, which predict that, in static reward environments such as dataset 1, the overall learning rate is diminished. To induce learning a dynamic decision-making task is required, such as dataset 2.

We also noted that we did not perform distribution decoding, whereby the reward distribution is decoded directly from neuronal activity^15^. Unlike in Dabney et al.^15^, the ground truth reward distribution in our dataset had a uniform shape, so it did not lend itself to qualitative comparison of multiple modes of the distribution.

#### Simultaneous diversity

Differences in value expectation may vary across sessions, owing to, for example, motivation. Therefore, when pooling neurons across sessions for analysis, we might find diversity even from classic RL alone owing to different expectations across sessions. To address this, we showed that diversity exists within single sessions (Extended Data Fig. 4). We also showed that diversity exists within individual subjects (Extended Data Fig. 4). We further accounted for possible diversity across subjects in the asymmetry predicting reversal point correlation: we found that the relationship between choice reversal point and feedback asymmetric scaling held after including subject as a coregressor (*t*(38) = 2.66, *P* = 0.01, for the asymmetric scaling regressor predicting reversal point, in a generalized linear model regressing out the subject). This suggests that differences in value expectations across subjects or sessions cannot explain the observed diversity.

### Dataset 2

#### Task and neural recordings —two-step decision task

Results in Fig. 3 are a subset of analyses from the neuronal recordings accompanying Miranda et al.^30^. A full report of the neurophysiological results during this task will be reported in upcoming separate publications. Full task details can be found in Miranda et al.^30^, but we outline the key points relevant to the present study here. As stated in Miranda et al.^30^, all experimental procedures were approved by the University College of London (UCL) Local Ethical Procedures Committee and the UK Home Office (PPL no. 70/8842) and carried out in accordance with the UK Animals (Scientific Procedures) Act.

This task was an adaptation of the classic two-step decision-making task^29^ to NHPs. The two-step nature of this task, along with the probabilistic transitions, is not relevant to the present study, because we focused analyses on the outcome time when the subjects were learning the values of the outcome stimuli in a manner that was postulated to be the same for model-based and model-free methods (Daw et al.^29^; see below). Nevertheless, we briefly describe the task here for completeness. Two decisions were made on each trial. At the first decision step, animals chose between two options (denoted by picture stimuli) which each resulted in probabilistic transitions to one of two second-stage states. One transition was more likely (70%, a common transition) and the other less likely (30%, a rare transition). The common transition from each of the first-stage options was to a different second-stage option. In each of the possible second stages, another two-option choice was required and each of these four end-stage states had one of three different outcome levels (high, medium and low reinforcement levels), which was delivered in the feedback stage. To induce learning, the outcome levels for the second-stage options were dynamic: reward associated with each second-stage option remained the same for five to nine trials, then changed randomly to any of the three possible outcome levels (including remaining the same). To make appropriate choices at both first and second stages of the task (which they did^30^), animals had to continually track and update the value of each end-stage stimulus.

We focused exclusively on neural activity at the feedback stage when outcome was received. This is because: (1) we wanted to focus on the learning of the dynamic values of the second-stage options to test for asymmetric learning; (2) it is at this feedback period when RPEs ought to be elicited and error-driven learning of option values occurs; and (3) this allows us to look at simple value learning independent of task transitions, which are not relevant for testing distributional RL. For the sake of our analyses, we could therefore think of this task as a simple reversal learning task in which four cues change their value every five to nine trials. Among other brain regions, recordings were made in the ACC. We focused analyses on the ACC because this is the brain region in common with Kennerley et al.^8^ and where we have a strong hypothesis for the presence of distributional RL.

#### Neurophysiological methods in the second (two-step) dataset

Two NHPs (subjects ‘J’ and ‘C’), different to those in Kennerley et al.^8^, performed the task. Subjects were two males aged 5–6 years, weighing 8–10 kg at the time of recording. Subjects were implanted with a titanium head positioner for restraint, then subsequently implanted with two recording chambers that were located on the basis of preoperative 3-T MRI and stereotactic measurements. Postoperatively, we used gadolinium-attenuated MRI and electrophysiological mapping of gyri and sulci to confirm chamber placement^19^. The chamber positioning along the AP, medial–lateral (ML) coordinate planes and their respective lateral tilt (LT) angle from vertical were as follows: one chamber over the left hemisphere at AP = 38(C)/37(J) mm, ML = 20.2(C)/18.1(J) mm and LT = 21°(C)/26°(J); and one over the right hemisphere at AP = 27(C)/27.5(J) mm, ML = 19.7(C)/17.9(J) mm and LT = 22.5°(C)/28°(J). Craniotomies were then performed inside each chamber to allow for neuronal recordings in different target regions.

For single-neuron recording we used epoxy-coated (FHC Instruments) or glass-coated (Alpha Omega Engineering) tungsten microelectrodes inserted through a stainless-steel guide tube mounted on a custom-designed plastic grid with 1-mm spacing between adjacent locations inside the recording chamber. Electrodes were acutely and slowly advanced through the intact dura at the beginning of every recording session using custom-built, micro-drive assemblies that were manually controlled and lowered electrodes in pairs or triplets from a single screw, or motorized microdrives (Flex MT and EPS by Alpha Omega Engineering) with individual digital control of electrodes. During a typical recording session, 8–24 electrodes were lowered into multiple target regions until well-isolated neurons were found. Neuronal signals were acquired at 40 kHz, amplified, filtered and digitized (OmniPlex Neural Data Acquisition System by Plexon Instruments). Spike waveform sorting was performed off-line using a principal component analysis-based method (Offline Sorter by Plexon Instruments). Channels were discarded if either neuronal waveforms could not be clearly separated or if waveforms did not remain stable throughout the session.

We randomly sampled neurons; no attempt was made to select neurons on the basis of responsiveness or specific cortical layer. This procedure ensured an unbiased estimate of neuronal activity, thereby allowing a fair comparison of neuronal properties between the different brain regions.

We recorded neuronal data from four target regions: the ACC, dorsolateral PFC (DLPFC), caudate and putamen. In subject C, we recorded from the ACC (dorsal bank of the ACC sulcus) and the DLPFC (dorsal bank of the principal sulcus) in both the left and the right hemispheres, and from the dorsal caudate and the dorsal putamen in the right hemisphere. In subject J, we recorded from the ACC (dorsal bank of the ACC sulcus) and the DLPFC (dorsal bank of the principal sulcus) in the left hemisphere; and from the dorsal caudate and the dorsal putamen from the right hemisphere. We recorded single-unit activity from 663 neurons (C: 695 and J: 246) in 57 recording sessions (C: 30 and J: 27) across all four investigated regions: ACC, 240 neurons; DLPFC, 187 neurons; caudate, 116 neurons; putamen, 120 neurons. We used gadolinium-enhanced MRI along with electrophysiological observations during the process of lowering each electrode to estimate the location of each recorded neuron. In the ACC, the recordings were positioned between AP 30–37 mm in subject C and AP 30–36 mm in subject J, relative to the interaural line (AP = 0 mm).

#### Neuron inclusion

Of the 240 neurons recorded in the ACC, we tested for signatures of distributional RL (see below) in those that were sensitive to RPE (those neurons that had *P* < 0.05 in linear regression between firing rate and RPE). The RPE regressors used to test for sensitivity are from Miranda et al.^30^. These were obtained using the best-fitting parameters fitted to behavior, as described in Miranda et al.^30^. Some 94 neurons passed this criterion and are the neurons analyzed in Fig. 3. Furthermore, we noted that the results held using a much more stringent definition of RPE from Bayer and Glimcher^31^ (Extended Data Fig. 8), that is, the firing rate at feedback on the current trial must be sensitive to the reward delivered on the current trial and on the previous trial, but with opposite signs, that is, ${{\mathrm{FR}}}={\beta }_{0}+{\beta }_{1}{{\mathrm{Rew}}}(t)+{\beta }_{2}{{\mathrm{Rew}}}(t-1)$, where *β*_1_ and *β*_2_ are both significant at *P* < 0.05 but with opposite signs; 33 neurons met this criterion. These are the neurons in which we analyzed the fit parameters, for testing for consistency and relationships between the parameters (Fig. 3f,g). Similar to dataset 1, the number of selective neurons in other regions was smaller than in the ACC (ACC: 94 of 240 = 39%; DLPFC: 39 of 187 = 21%; caudate: 26 of 115 = 23%; putamen: 34 of 119 = 29%; Extended Data Fig. 7). Furthermore, there were too few neurons selective under the aforementioned stringent definition of RPE^31^ for further model comparisons (Extended Data Fig. 7), and thus we focused our analyses on ACC.

### Models and model fitting to test for asymmetric learning

#### Models

To test for asymmetric learning, we modeled neuron responses with classic and distributional RL models and tested which was a better fit to the data. In all cases the model was used, for each neuron, to predict the firing rate on each trial (mean firing rate in a window of 200–600 ms after feedback).

We adapted the one-step transition temporal difference learning model wherein estimates of cue values *V* are updated according to:

2 $$V\leftarrow V+\alpha \delta$$

where *δ* is the RPE, *δ* = *r* − *V*, with *r* the reward delivered on the current trial and *V* the previous value estimate, and *α* is the learning rate by which *δ* is scaled to update values. This is the equation for classic RL and amounts to the Rescorla–Wagner model^10^.

The distributional RL version of this model is^15^:

3 $$\begin{array}{c}V\leftarrow V+{\alpha }^{+}\delta ,\delta > 0\\ V\leftarrow V+{\alpha }^{-}\delta ,\delta \le 0\end{array}$$

where *α*^+^ and *α*^−^ are separate learning rates for positive and negative RPEs/*δ*. In other words, the learning rate associated with a value update on a given trial will depend on whether the RPE was positive or negative. Different learning rates for positive and negative RPEs result in asymmetries in the rates at which neurons learn from better-than-expected and worse-than-expected feedback, that is, asymmetric learning. This is unlike classic RL where learning is symmetric.

To fit the model to neural data, we predicted the firing rate at feedback from the RPE. For the classic RL case this was as follows:

4 $${{\mathrm{FR}}}={\beta }_{0}+{\beta }_{1}\delta$$

where *β*_0_ and *β*_1_ are regression coefficients. In the distributional RL case we have:

5 $$\begin{array}{c}{\mathrm{FR}}={\beta }_{0}+{\beta }^{+}\delta ,\delta > 0\\ {\mathrm{FR}}={\beta }_{0}+{\beta }^{-}\delta ,\delta \le 0\end{array}$$

where *β*^+^ and *β*^−^ are different regression coefficients for positive and negative RPEs/*δ*; that is, allows the FR to be a different scaling of the RPE for positive and negative RPEs. Critically, in these models, this asymmetric scaling is separable from the above asymmetric learning, because it does not directly impact the update of the cue value *V*, and therefore subsequent computation of RPEs (*r* − *V*). This allowed us to isolate learning and scaling effects from each other and therefore separately measure them and demonstrate their existence (see below). Hence, it is possible to measure asymmetric scaling without asymmetric learning and vice versa. Both asymmetric scaling and asymmetric learning are predictions of the distributional RL theory, as is a relationship between the two. Asymmetric scaling is what was tested for in Dabney et al.^15^ (it was not possible to test for learning dynamics in the task they analyzed, nor the first dataset in this paper, owing to the static nature of cue values).

We designed our models such that asymmetric scaling did not impact the update of the value so that we could estimate asymmetric scaling and asymmetric learning separately in the model. The beta parameters scaled the prediction error to predict neural firing rate. We measured the downstream effects of the prediction error on learning (that is, value updating) via the alpha parameters. The advantage of this separation is that it allowed us to isolate the asymmetric learning effect to show that it truly exists. An earlier model where the asymmetric scaling did impact the value updating (that is, ${\beta }^{+}={\alpha }^{+}$ and ${\beta }^{-}={\alpha }^{-}$) outperformed the symmetric model; however, we realized that this could outperform the symmetric model due to the presence of asymmetric scaling alone, without asymmetric learning, risking a false-positive conclusion that there was asymmetric learning. We concluded that, to get reliable evidence of the asymmetric learning effect (that is, asymmetric value updates), we had to model them separately to isolate the learning effect. Note, therefore, that we were not modeling them separately because we did not think that they were related. On the contrary, an additional reason for estimating asymmetric scaling and asymmetric learning separately in the model was that it allowed us to correlate the parameters and conclude that there was indeed a relationship between them (Fig. 3g), as predicted by distributional RL.

We therefore had four possible models to test: symmetric learning and symmetric scaling (‘fully classic RL’; SLSS), symmetric learning and asymmetric scaling (SLAS), asymmetric learning and symmetric scaling (ALSS) and asymmetric learning and asymmetric scaling (‘fully distributional RL’, ALAS).

#### Model fitting

We tested which of the above models were the best fit to the data. We did this by fitting the parameters in a subset of the data and tested how well (measured using *R*^2^) a model using these fit parameters explained the held-out data in a tenfold crossvalidation procedure. We then asked which model was the best fit to the data.

#### Fitting a simplified, single asymmetric scaling parameter

As fitting all four parameters to the data was computationally demanding, we adapted the asymmetric scaling equations such that asymmetric scaling could be accounted for with one, rather than two, parameters. We replaced the asymmetric scaling equations with the following:

6 $$\begin{array}{c}{\mathrm{FR}}={\beta }_{0}+{\beta }_{1}\delta S,\delta > 0\\ {\mathrm{FR}}={\beta }_{0}+{\beta }_{1}\delta (1-S),\delta \le 0\end{array}$$

where *S* is bounded between 0 and 1 and acts as a single asymmetric scaling parameter (for example, if *S* is near 1, positive RPEs are scaled greatly relative to negative RPEs). Using *S* rather than fitting *β*^+^ and *β*^−^ therefore still achieves the important effect of accounting for asymmetries in the scaling of the FR by positive versus negative RPEs. This can be understood by the following: the asymmetry in scaling is represented by the ratio ${\beta }^{+}/({\beta }^{+}+{\beta }^{-})$; substituting in *β*^+^ = *S* and ${\beta }^{-}=1-S$, we have: ${{\mathrm{Asymmetric}}\; {\mathrm{scaling}}}=\frac{{\beta }^{+}}{{\beta }^{+}+{\beta }^{-}}=\frac{S}{S+\left(1-S\right)}=S$. Therefore the *S* parameter is equal to the asymmetry in scaling. Note that the regression coefficients, *β*_0_ and *β*_1_, are the same in both equations, that is, they were fitted in the same regression model (positive and negative RPE trials were included in the same regression model), having scaled the RPEs by *S* or 1 − *S*. Also note that it is the scaling parameter, *S*, which captures the asymmetry, that is, trained and tested in crossvalidation, not the beta values (see below). It is also this parameter that is used as a measure of asymmetric scaling that is correlated with asymmetric learning in Fig. 3.

#### Estimating the parameters

We generated RPE regressors from each of the models and regressed these against neural data. The regressors were generated by passing through the model the option chosen and reward observed on each trial of the training set. Values were updated and RPEs computed on each trial according to the above equations. We measured the model fit using the *R*^2^ value computed from the regression model. For each model (for example, asymmetric learning with asymmetric scaling) we carried out the model fitting using a grid search over parameter space. Possible values for each parameter that is fitted to the data—*α*^+^, *α*^−^ and *S*—lie between 0 and 1, and we performed the grid search with 0.025 size increments (this is an additional advantage to using *S* rather than *β*^+^ and *β*^−^, because the former but not the latter is bounded by 0 and 1, and can therefore be more easily fitted using a grid search). The combination of parameters with the highest *R*^2^ value was taken to be the best fit of parameters to the data. The linear parameters *β*_0_ and *β*_1_ were estimated on each grid search using linear regression.

#### Testing in held-out data

For a given model, we took this combination of best-fitting parameters and used them to generate regressors in the held-out data with the model equations above, again using the option chosen and reward delivered on each trial. We then assessed their fit to the data by regressing the RPEs computed by the model in these held-out trials against the firing rates on those trials. This resulted in *R*^2^ values for the held-out data, dependent on parameters fit to the training data, such that parameters capturing features of the data consistent across crossvalidation folds would result in better fits in the held-out data. We obtained ten *R*^2^ values for each model for each neuron; one for each crossvalidation fold.

In Fig. 3, the linear parameters *β*_0_ and *β*_1_ are refitted in the held-out test data. This is because the linear parameters capture and can remove variance in which we are not interested for our main asymmetry analyses, for example, if a given fold happened to have an increase in the overall gain that is not related to the asymmetry. Re-estimating the linear parameters isolated the model comparison to our effect of interest, that is, the asymmetry effects. However, we also showed that the results are qualitatively and quantitatively very similar if we carried the linear parameters over data partitions, that is, did not re-estimate them, but rather used, the linear parameters fitted in the training data to predict the firing rates in the held-out test data (Extended Data Fig. 9).

#### Testing for differences between model fits

We then compared, across the population of neurons, the different models’ fits to the neural data. We took the mean across the ten crossvalidated model fits in the test data for each model for each neuron, giving one number per model per neuron. We then carried out paired Student’s *t*-tests between the different models to determine the best-fitting model. We found that the asymmetric scaling with asymmetric learning model was better than all other models. This meant that the extra parameters improved the explanation of the neural data in the held-out data (despite having to fit more parameters to the data), demonstrating that asymmetric learning is a better account of the data than symmetric learning.

Note that, although we focused on model comparisons, the absolute goodness of fits (*R*^2^) for each model were as follows: ALAS = 0.1332 ± 0.0098 (mean ± s.e.m.); SLAS = 0.1277 ± 0.0096; ALSS = 0.1210 ± 0.0091; and SLSS = 0.1184 ± 0.0090.

### Statistics and reproducibility

The sample sizes were chosen to be two animals for each of dataset 1 and dataset 2, as discussed in the supporting references^8,30^. Two animals per dataset is the commonly used number for macaque studies and is standard across virtually all macaque electrophysiology studies. No data were excluded from the analyses, except neurons that did not meet the criterion (for example, as RPE neurons) to enter the analyses, as discussed above. On the note of reproducibility, we would like to point out that we found evidence for distributional RL across two independent datasets. Statistics were conducted using MATLAB 2019a. Data distribution was assumed to be normal. Where relevant, trials and transitions between task contingencies were randomized in the task design.

### Reporting summary

Further information on research design is available in the Nature Portfolio Reporting Summary linked to this article.

## Online content

Any methods, additional references, Nature Portfolio reporting summaries, source data, extended data, supplementary information, acknowledgements, peer review information; details of author contributions and competing interests; and statements of data and code availability are available at 10.1038/s41593-023-01535-w.

### Supplementary information

Supplementary Information Supplementary Discussion and Supplementary Fig. 1.

Reporting Summary

## Data Availability

The present study performs a re-analysis of a previously published neural data^8^ and presents the neural data results from a second dataset that reported only behavior and computational modeling^30^. Data availability will be in line with those primary source studies. Dataset 2 (Miranda et al.^30^) will be shared in an upcoming separate publication.

## Code availability

Code is available upon request.
