# Supplementary material for: Distributional reinforcement learning in prefrontal cortex
Source: Nat Neurosci. 2024 Jan 10;27(3):403–8. doi: 10.1038/s41593-023-01535-w (PMC10917656; doi:10.1038/s41593-023-01535-w)
Supplement: Supplementary file 1 — Supplementary Discussion and Supplementary Fig. 1. [file 41593_2023_1535_MOESM1_ESM.pdf]

nature neuroscience

Brief Communication

https://doi.org/10.1038/s41593-023-01535-w

Distributional reinforcement learning in prefrontal cortex

In the format provided by the authors and unedited

Supplementary discussion

Possible future task:

Although the presented datasets allowed us to test several key predictions of distributional RL theory and extend the original findings of Dabney, Kurth-Nelson et al 2020[1], all of these experiments were not originally designed to specifically examine distributional RL. Here we describe an experiment for testing further distributional RL predictions; specifically how different reward distributions may be represented. It would be informative if recordings from such an experiment had data from both dopaminergic and PFC neurons to examine similarities and differences. Furthermore, one could exploit the anatomical gradient we have reported in the current study to silence optimistic or pessimistic subregions selectively (with, for example, transcranial focused ultrasound stimulation) and examine effects on the neural representation of the distribution and/or behaviour.

This proposed experiment (Supplementary Figure 1) would allow us to examine how a population of neurons represents multiple different reward distributions. Distributional RL predicts the neuronal representation of different reward distributions should be different, even when the distributions are matched for summary statistics such as the mean and variance. On each trial, subjects are presented with a cue, followed by a reward of varying magnitude. Each cue is predictive of a different reward distribution (Supplementary Figure 1A). Importantly, each distribution has a partner that is matched for mean and variance but different in higher moments of the distribution.

Furthermore, the presentation of such cued distributions could be embedded in a two-alternative forced choice task that encourages the use of such modes of the distribution by manipulating risk pressures[2] (Supplementary Figure 1B-C). In the two-alternative forced choice task two options are presented on a given trial and the subject must choose between them; this task may be augmented such that the use of different modes of the reward distributions is required. This could be achieved by having trials occurring in blocks, and within each block of trials there is a required minimum amount of reward to be obtained; if it is not obtained, no overall reward will be delivered for that block of trials (Supplementary Figure 1B). This induces different “risk pressures” in different blocks, as near the end of a high pressure block, if the threshold has not yet been reached, subjects must choose higher risk, higher reward options, such as those with a low probability but high reward mode of the distribution (Supplementary Figure 1D). This will encourage use of such modes of the distribution.

Neurally, these tasks allow an experimenter to test for different representations of the options (Supplementary Figure 1E-F), even if they are matched for summary statistics. Unlike classic RL, distributional RL would predict that any two options that have the same expectation, but different underlying distributions would have different neural representation, and would predict a different pattern of RPEs at reward delivery, depending on the cued distribution (Supplementary Figure 1E-F).

References

1. Dabney, W. *et al.* A distributional code for value in dopamine- based reinforcement learning. *Nature* **577**, 671–675 (2020).
2. Kolling, N., Wittmann, M. & Rushworth, M. F. S. Multiple neural mechanisms of decision making and their competition under changing risk pressure. *Neuron* **81**, 1190–1202 (2014).

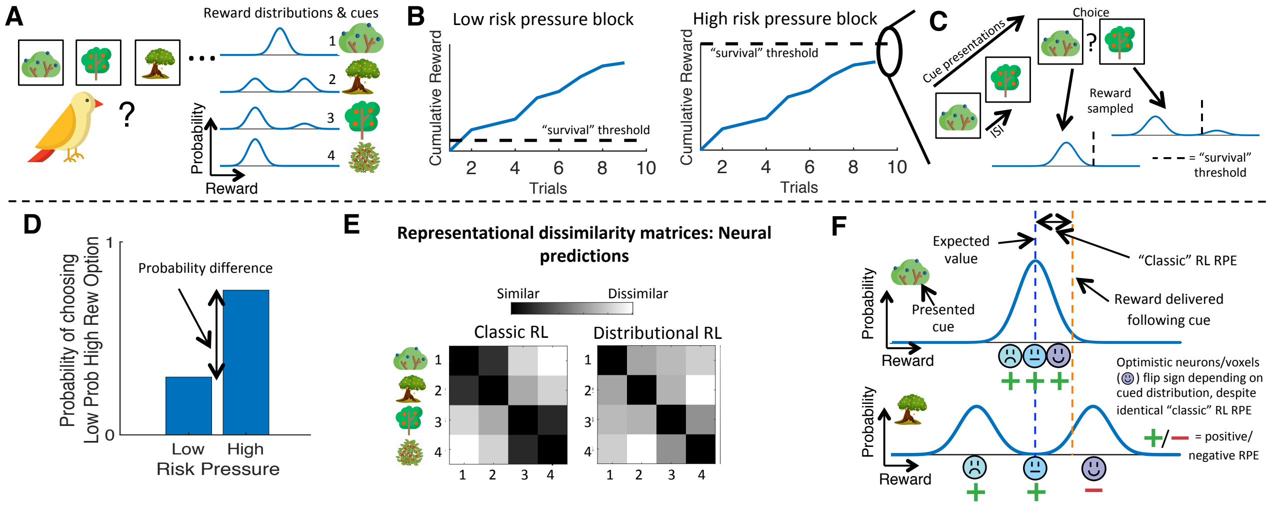

Supplementary Figure 1 – A possible future task and experiment: Paradigm probing the neural representation of reward distributions, along with predictions. Note that everything in this figure is from simulation or predicted effects; there are no real data. We are presenting a possible task to test for distributional representations. A-D) A possible dynamic risk pressure task with distributions. **A)** Subjects choose between different cues, e.g. they control a bird obtaining reward from different cues (trees). On each trial, a cue is chosen, and each cue gives rise to reward sampled from different distributions. Distributions can be of unusual shape (e.g. bimodal) and matched for summary statistics (e.g. expected value, variance). **B)** Trials occur in blocks, in which the subject accumulates reward over trials, but only gets to keep it if a certain reward level (black line/"survival threshold") is reached. Varying this reward level applies varying levels of pressure to make risky choices, inducing participants to have dynamically shifting risk preferences (e.g. as in Kolling et al 2014). **C)** These dynamic risk preferences force participants to use the reward distributions in their choices. This is because choosing the highest expected value option will often not be the best choice, since you may need to choose a distribution with a low probability of high reward to reach the threshold reward level. **D)** Predicted behavioural effect: As in C, when risk pressure is high, participants should choose low probability, high reward options. **E)** Neural prediction: Distributional RL predicts cues with different underlying reward distributions should be represented differently, even if their expectation (and variance) are the same (unlike in classic RL). **F)** Another neural prediction is that some neurons (or voxels) will have opposite signed effects following the same reward delivered after a cue with the same expected value, depending on the distribution, even though classic RL RPEs are identical.
